# Supplementary material for: Hybrid Models and Biological Model Reduction with PyDSTool
Source: PLoS Comput Biol. 2012 Aug 9;8(8):e1002628. doi: 10.1371/journal.pcbi.1002628 (PMC3415397; doi:10.1371/journal.pcbi.1002628)
Supplement: Text S4 — Complete source code for the PyDSTool package (version 0.88.120504). Includes API documentation and help files linking to web pages. This file is identical to the current public release on Sourceforge.net. (ZIP) [file pcbi.1002628.s004.zip › PyDSTool/html/PyDSTool.Toolbox.ParamEst.ParamEst-class.html]

xml version="1.0" encoding="ascii"?


PyDSTool.Toolbox.ParamEst.ParamEst


| Home | Trees | Indices | Help | PyDSTool |
| --- | --- | --- | --- | --- |

Package PyDSTool ::
Package Toolbox ::
Module ParamEst ::
Class ParamEst

[hide private]

[frames] | no frames]

# Class ParamEst

source code

```
    object --+    
             |    
common.Utility --+
                 |
                ParamEst
```

Known Subclasses:
:   - BoundMin
    - , LMpest

---

General-purpose parameter estimation class. freeParams keyword
initialization argument may be a list of names or a dictionary of scales
for determining appropriate step sizes for O(1) changes in the residual
function.

In its absence, the scales will default to 1.


|  |  |
| --- | --- |
| Instance Methods | [hide private] |

|  |  |
| --- | --- |
| \_\_init\_\_(self, \*\*kw)  x.\_\_init\_\_(...) initializes x; see x.\_\_class\_\_.\_\_doc\_\_ for signature | source code |

|  |  |
| --- | --- |
| resetParArgs(self) | source code |

|  |  |
| --- | --- |
| setAlgParams(self, \*args)  Set algorithmic parameters. | source code |

|  |  |
| --- | --- |
| setFn(self, fn) | source code |

|  |  |
| --- | --- |
| evaluate(self, extra\_record\_info=None)  Evaluate residual vector, record result, and display step information (if verbose). | source code |

|  |  |
| --- | --- |
| reset\_log(self) | source code |

|  |  |
| --- | --- |
| key\_logged\_residual(self, pars\_ics, weights)  pars\_ics must be a sequence type | source code |

|  |  |
| --- | --- |
| find\_logs(self, res\_val=None, condition=`'``<``'`)  Find log entries matching given condition on their residual norm values. | source code |

|  |  |
| --- | --- |
| show\_log\_record(self, i, full=False)  Use full option to show residuals mapped to their feature names, including information about weights. | source code |

|  |  |
| --- | --- |
| pars\_to\_ixs(self) | source code |

|  |  |
| --- | --- |
| pars\_array\_to\_dict(self, parray) | source code |

|  |  |
| --- | --- |
| pars\_dict\_to\_array(self, pdict) | source code |

|  |  |
| --- | --- |
| par\_sensitivity(self, pdict=None, non\_diff\_feats=None, extra\_info=False)  Parameter sensitivity of the context's features at the free parameter values given as a dictionary or args. | source code |

|  |  |
| --- | --- |
| weighted\_par\_sensitivity(self, feat\_sens)  Return parameter sensitivities weighted according to current feature weights, based on a previous output from par\_sensitivity method. | source code |

|  |  |
| --- | --- |
| run(self)  Run parameter estimation. | source code |

|  |  |
| --- | --- |
| iterate(self) | source code |

**Inherited from `object`**:
`__delattr__`,
`__getattribute__`,
`__hash__`,
`__new__`,
`__reduce__`,
`__reduce_ex__`,
`__repr__`,
`__setattr__`,
`__str__`


|  |  |
| --- | --- |
| Properties | [hide private] |

**Inherited from `object`**:
`__class__`


|  |  |
| --- | --- |
| Method Details | [hide private] |

|  |  |
| --- | --- |
| \_\_init\_\_(self, \*\*kw)  *(Constructor)* | source code |

x.\_\_init\_\_(...) initializes x; see x.\_\_class\_\_.\_\_doc\_\_ for
signature

Overrides: object.\_\_init\_\_: *(inherited documentation)*

|  |  |
| --- | --- |
| find\_logs(self, res\_val=None, condition=`'``<``'`) | source code |

Find log entries matching given condition on their residual norm
values. Returns a list of log indices.

if res\_val is not given, the residual norm of the first entry in the
current log is used.

Use '<' and '>' for the condition argument (default is
<).

|  |  |
| --- | --- |
| par\_sensitivity(self, pdict=None, non\_diff\_feats=None, extra\_info=False) | source code |

```
Parameter sensitivity of the context's features at the free parameter
values given as a dictionary or args. If none provided, the current
test model parameter values will be used. A dictionary mapping parameter names to
  {interface_instance: {feat1: sensitivity_array, ..., featn: sensitivity_array}}
is returned.

Specify any non-differentiable features in the non_diff_feats list
as pairs (interface instance, feature instance).

Sensitivity entry > 0 means that increasing the parameter will
increase the absolute value of that residual, i.e. worsen the "fit".

extra_info optional argument makes this method return both the feature sensitivity
dictionary and a dictionary containing additional information to reconstruct
the gradient of the residual norm, to save re-calculation of it at this point.
This gradient will also respect the non_diff_feats argument, if provided.
```

|  |  |
| --- | --- |
| run(self) | source code |

Run parameter estimation. Returns a dictionary:

'success' -> boolean 'pars\_sol' -> fitted values of pars
'pars\_orig' -> original values of optimized pars 'sys\_sol' ->
trajectory of best fit Model trajectory 'alg\_results' -> all other
algorithm information (list)

  


| Home | Trees | Indices | Help | PyDSTool |
| --- | --- | --- | --- | --- |

|  |  |
| --- | --- |
| Generated by Epydoc 3.0.1 on Fri May 4 15:24:08 2012 | http://epydoc.sourceforge.net |
